# Evaluation of the MYNX CONTROL™ Arterial Closure System for Achieving Primary Hemostasis after Arterial Femoral Access Following Peripheral Arterial Interventions, Compared to the FemoSeal^TM^ Closure System

**DOI:** 10.3390/jcm12165255

**Published:** 2023-08-12

**Authors:** Elias Noory, Tanja Böhme, Leonie Krause, Philipp Ruile, Jonas Salm, Ulrich Beschorner, Roaa Bollenbacher, Dirk Westermann, Thomas Zeller

**Affiliations:** Department of Cardiology and Angiology, Medical Center—University of Freiburg, 79189 Bad Krozingen, Germany

**Keywords:** femoral access, vascular closure device, complication, FemoSeal, MYNX

## Abstract

**Background:** In addition to manual compression, various vascular closure devices (VCD) are available to seal the puncture site following arterial vascular procedures. **Purpose:** To evaluate the efficacy and safety of the extravascular MYNX CONTROL closure system for achieving primary hemostasis after femoral arterial access following peripheral arterial procedures, compared to the intravascular FemoSeal Aclosure system. **Patients and Methods:** A retrospective analysis of consecutive patients who underwent endovascular intervention between April and November 2022 was performed. The primary endpoint was the incidence of significant puncture site complication defined as a complication resulting in medical treatment. Secondary endpoints included peri-interventional incidence of hematoma, peri-interventional changes in hemoglobin, incidence of emergency diagnostics and predictors for closure system failure. **Results:** Five hundred and forty-eight patients were included in this analysis. False aneurysm occurred in 18/273 cases (6.6%) following the use of the MYNX closure system, compared to 6/275 cases after using the FemoSeal closure system (2.2%, *p* = 0.006). The incidence of post-interventional hematoma was not significantly different (28 (10.3%) in the MYNX group versus 32 (11.6%) in the FemoSeal group, *p* = 0.358). Peri-interventional hemoglobin drop did not differ between groups (*p* = 0.449). Emergency diagnostics were not significantly performed more often in the MYNX group (14 (5.1%) versus 8 (2.9%), *p* = 0.134). A post-interventional duplex sonography showed stenosis at the puncture site in one patient after use of the MYNX system. For the entire cohort, oral anticoagulation was the only predictor for the failure of the closure device (*p* = 0.036). **Conclusions:** Device failure was more common after using the extravascular MYNX CONTROL system than after using the intravascular FemoSeal system. However, the need for surgical or interventional therapy due to device failure was low.

## 1. Introduction

In addition to manual compression, various vascular closure devices (VCD) are available for post-interventional management of the puncture site after arterial vascular intervention, and they induce rapid or immediate hemostasis at the puncture site.

The FemoSeal™ closure system (Terumo Medical Corporation, Leuven, Belgium) is an intravascular closure system. It consists of two absorbable polymer discs and an absorbable suture. The puncture site is compressed using the two plates in the “sandwich principle” and is thus closed [1]. Access-site-related vascular complications are less frequent after using the FemoSeal closure system than after manual compression [2,3].

The MYNX CONTROL closure system (CardinalHealth, Dublin, OH, USA) is an extravascular collagen-based closure system. For this, a semi-compliant balloon is positioned in the vessel, and Polyethylene glycol sealant is released outside the vessel [4]. The MYNX CONTROL closure system is identical to the MYNXGRIP system, except that the MYNX CONTROL system is easier to handle and place. Therefore, MYNX CONTROL has replaced MYNXGRIP as the succeeding product.

A randomized, single-centre study showed fewer major adverse vascular events with the MYNXGRIP system than with manual compression, and the time to hemostasis and mobilization was shorter with the MYNXGRIP system [5]. Further studies have demonstrated the safety and efficacy of this extravascular closure system. [6,7].

A study showed that closure device failure was lower in the intravascular VCD group, compared with the extravascular VCD group [8].

The purpose of this retrospective study, which includes consecutive patients, is to evaluate the efficacy and safety of the MYNX CONTROL closure system for achieving primary hemostasis after arterial femoral access following peripheral arterial interventions, compared to the FemoSeal closure system. The hypothesis is that compared to the FemoSeal system, hemostasis after a peripheral intervention is also safely achieved with the MYNX Control system.

## 2. Methods

### 2.1. Patient Population

Patients who received a femoral puncture for the endovascular treatment of peripheral arterial disease at a university high-volume centre were selected. Consecutive patients treated between April and November 2022 were included in the analysis. The local ethics committee provided study approval (7 February 2023). The only inclusion criterion was the use of the MYNX CONTROL or the FemoSeal closure system. Accordingly, the use of a different closure system or a vessel closure only via manual compression were exclusion criteria. Diagnostic angiographies were excluded. All procedures were peripheral interventions.

### 2.2. Study Endpoints

The primary endpoint of the study was the incidence of significant puncture site complication defined as a complication resulting in medical treatment (surgery, intervention, ultrasound-guided compression, thrombin injection or administration of blood products for puncture site bleeding). Both the post-interventional incidence of a pseudoaneurysm and continuous bleeding from the puncture site after the use of the device in the catheter laboratory, with the need for further hemostatic interventions, were summarized as device failures. Secondary endpoints were peri-interventional hemoglobin drop, incidence of emergency diagnostic procedures and predictors for closure system failure.

To assess complications such as hematoma, swelling and bleeding at the vascular access site, the site was visually inspected. Duplex ultrasound was performed on all patients on the day after the procedure. The patients were all treated as inpatients and monitored for at least 24 h. The length of inpatient stay depended on comorbidities, the need for amputations and the complexity of the endovascular procedure.

### 2.3. Endovascular Procedure

Femoral artery access was obtained by direct puncture using the Seldinger technique. Intervention details resulted from the angiographic findings and the investigator’s procedure.

At the end of the procedure, different closure systems were available for closing the femoral access. The decision for a closure system was made by the interventionalist.

The FemoSeal closure system (Terumo Medical Corporation, Leuven, Belgium) is an intravascular closure system. It consists of two absorbable polymer discs and an absorbable suture. After removal of the sheath, the system is inserted via a guide wire.

One of the polymer plates is placed on the inner wall of the vessel as an anchor. On the outer wall of the vessel, the other polymer disc serves as a securement. The puncture site is compressed using the two plates in the “sandwich principle” and is thus closed [1].

The MYNX CONTROL closure system (CardinalHealth, Dublin, OH, USA) is an extravascular collagen-based closure system. For this, a semi-compliant balloon is positioned in the vessel, and Polyethylene glycol sealant is released outside the vessel [4].

The MYNX CONTROL closure device was deployed in the standard manner as instructed by the device manufacturer. At first, the device was used exclusively under the observation of a company representative.

Depending on the examiner’s decision, a pressure bandage was also applied.

Both pre-existing antithrombotic therapy or anticoagulation and the administration of a loading dose were noted. Heparin was used during the procedure, and the dose was at the operator’s discretion.

In both groups, bed rest for six hours after the closure procedure was prescribed.

Before hospital discharge, routine duplex sonography of the access site was performed.

## 3. Statistical Analysis

Statistical analysis was performed using SPSS software (version 25.0; SPSS, Chicago, IL, USA). Continuous data are presented as means ± standard deviation; categorical data are given as counts (percentages). Categorical variables were compared with Fisher’s exact test, and continuous data were compared with the Student’s t-test. Statistical significance was accepted at *p* < 0.05. Binary logistic regression analysis with stepwise forward variable selection was used to identify risk factors for the failure of the closure device. The following patient characteristics and treatment details were included in the analysis: age, body mass index, comorbidities, smoking status, blood thinning medication, intervention details and calcification (at least 30% eccentric or concentric calcification).

## 4. Results

Overall, 548 patients were included in this analysis, *n* = 273 in the MYNX CONTROL cohort and *n* = 275 in the FemoSeal cohort, respectively. Hypertension and hyperlipidemia were the most common cardiovascular risk factors in both groups. The baseline characteristics are shown in Table 1. Patients treated with the MYNX CONTROL closure system were more likely to have coronary heart disease (47.3% versus 39.6%, *p* = 0.043).

The pre-interventional laboratory and blood pressure values for both groups are presented in Table 2. There were no significant differences in pre-interventional blood thinning medication between the groups (Table 3).

The MYNX CONTROL closure system was used more frequently after an antegrade puncture. The FemoSeal system was used more frequently after lysis. Puncture and intervention details are shown in Table 4. The MYNX CONTROL closure system failed to achieve hemostasis in 21 cases (7.7%). In contrast, the FemoSeal closure system failed in 11 cases (4.0%) (*p* = 0.047).

Pseudoaneurysm occurred in 18 cases (6.6%) following the use of the MYNX closure system and in six patients (2.2%) after using the FemoSeal closure system (*p* = 0.006). Thirteen of the pseudoaneurysms in the MYNX group were treated with manual compression. One pseudoaneurysm was treated via compression and thrombin injection, two by thrombin injection and two with endovascular treatment.

In the FemoSeal group, manual compression was performed in three cases, and in one case it was treated with a thrombin injection. One aneurysm was operated on, and one was endovascularly treated.

Post-interventional duplex sonography showed stenosis at the puncture site in one patient after use of the MYNX CONTROL system. The stenosis could be treated conservatively.

Peri-interventional hemoglobin drop (1.15 ± 1.1 g/dL in the MYNX group versus 1.16 ± 1.6 g/dL in the FemoSeal group, *p* = 0.449) did not differ between groups.

By trend, emergency diagnostic procedures were performed more often in the MYNX CONTROL group (14 (5.1%) versus 8 (2.9%), *p* = 0.134).

For the MYNX cohort, oral anticoagulation was the only predictor for the failure of the closure device (*p* = 0.036), Table 5. In the FemoSeal group, the use of platelet aggregation inhibitors was predictive of the failure of the closure device (*p* = 0.020), Table 6.

## 5. Discussion

The common femoral artery is the most common vascular access site for peripheral or cardiac interventions [9]. Numerous studies investigating closure systems have been performed after diagnostic coronary angiography. Complications, including access site complications, in therapeutic interventions are higher [10].

In the present work, two closure devices were investigated after peripheral intervention, not after diagnostic angiography. The MYNX CONTROL system significantly failed to achieve hemostasis more frequently, compared to the FemoSeal (7.7% versus 4.0%; *p* = 0.047). In addition, pseudoaneurysms also significantly occurred more frequently (6.6% versus 2.2%, *p* = 0.006) in the MYNX CONTROL cohort.

The reported device failure rate varies widely and is difficult to compare due to the different complication definitions.

After diagnostic coronary angiography, Schulz-Schüpke et al. showed that VCD was associated with a lower rate of hematomas than manual compression (4.8% versus 6.8%), and closure device failure was lower with the intravascular VCD group, compared with the extravascular VCD group (5.3% versus 12.2%) [8].

Nevertheless, other studies have shown the safety and effectiveness of extravascular closure systems so that the superiority of intravascular closure systems cannot be conclusively assessed.

In another study, 190 patients were enrolled (95 diagnostic cases and 95 interventional cases). One (0.5%) vascular complication (transfusion) occurred in one patient. No complications attributable to the occlusion system that were associated with serious clinical sequelae were reported [6].

Diamantopoulos et al. [7] demonstrated the effectiveness and safety of the MYNX CONTROL system in 100 patients in the first prospective study. Immediate hemostasis was achieved in 97% of cases, only three patients experienced a pseudoaneurysm, and one patient had a small hematoma. No clinically serious sequelae have been reported after the use of the MYNX CONTROL System up to 30 days after the index procedure. In this study, minor complications were more frequent after antegrade puncture than after retrograde puncture.

In the present work, the MYNX CONTROL system was used significantly more often after the antegrade puncture, which could explain the slightly higher failure rate.

Overall, the study by Diamantopoulos et al. and the present work are difficult to compare because, on the one hand, the endpoints were defined differently, and in Diamantopoulos et al., non-exclusive peripheral interventions were the indication for the puncture. It is likely that the common femoral artery was more severely altered in patients with a peripheral artery disease than in patients undergoing, for example, prostate artery embolization.

In the retrospective study of Fields et al., a pseudoaneurysm occurred in three patients (11%) [11].

In addition to bleeding complications, the use of the closure system can also lead to stenosis through the sealant of the closure device. In this study, this was the case for one patient in the MYNX CONTROL group (0.4%). Fields et al. stated that sealant material was found in the artery in 5 of 27 cases (18%) after using the MYNX device [11]. Although it did not occur in this study, stenosis can also occur after the use of an intravascular closure system, for example, if a polymer disc does not attach properly to the vessel wall.

The indication for emergency diagnostic procedures (usually colour duplex sonography) was given more frequently in the MYNX CONTROL group. It can be assumed that the indication for diagnostic procedures was made more quickly in the MYNX CONTROL group, as the experience here was not yet as pronounced as with FemoSeal, which had been used for a long time. Another explanation could also be that the MYNX CONTROL system was used more often after antegrade intervention, and bleeding was more difficult to detect here.

A small decrease in hemoglobin was observed peri-interventionally in both groups. However, there was no significant difference between the groups. Hemoglobin drop has not been evaluated in other studies [5,6,11]. A drop in hemoglobin can also occur peri-interventionally, for example, in the case of bleeding along the sheath or when using a thrombectomy system. It is not exclusively caused by bleeding at the puncture site and thus cannot be attributed to the closure device.

Whether the risk of complications due to vessel wall changes, for example, calcification in patients with PAD has not been conclusively clarified.

One study showed that calcification, particularly the presence of calcification in the anterior femoral artery, was the most important determinant of failure of the Perclose ProGlide and Prostar XL (Abbott Vascular, Redwood City, CA, USA) after endovascular abdominal aortic aneurysm repair [12]. After peripheral interventions, another study showed a success rate of the occlusion system independent of calcification [13]. Even after antegrade application of the FemoSeal system, calcification was not a predictor of complications [14].

In the present work, calcification was not significantly different between the device groups, and calcification was not a predictor of failure.

Unlike in the study by Scott et al., in the present study, oral anticoagulation was a predictor of MYNX closure system failure [15].

In another study, the outcome after coronary angiography was investigated. Patients with and without oral anticoagulation were compared. Patients on oral anticoagulation did not have higher 30-day major adverse events (major bleeding, vascular complications or cardiovascular-related death) than those who were not anticoagulated at the index procedure [16].

Nevertheless, increased caution should be exercised during femoral puncture and peri-interventional management in patients on oral anticoagulation.

The only predictor of FemoSeal failure was the long-term use of a platelet aggregation inhibitor. This cannot be explained and must be interpreted with caution due to the small number of patients. In the MIRROR study, which investigated single (aspirin) or dual antiplatelet therapy (aspirin and clopidogrel) after the peripheral intervention, there were no more complications at the access site under the dual therapy regime [17].

In the present study, sheath size was not investigated as a potential risk factor because a 6-French sheath was predominantly used, and only in individual cases, a larger (*n* = 16) or smaller sheath (*n* = 17) was used.

However, studies have shown a correlation between sheath size and complications at the access site. Cacuci et al. studied the incidence of access complications after endovascular procedures (*n* = 5385). Complications occurred in 16.6%, and an independent predictor of such a complication was, among others, the sheath size (OR per French, 1.59; *p* < 0.001) [18]. Kalish et al. also found that a sheath size greater than 6 French was a predictor of groin hematomas after peripheral vascular intervention (OR, 1.62; 95% CI, 1.24–2.11 and *p* < 0.001) [19].

## 6. Limitations

This is a retrospective, non-randomized study, which means that some selection bias cannot be excluded. The fact that the examiners have been using the FemoSeal system for years and have not been familiar with the MYNX CONTROL system for so long can also influence the results.

Another limitation of the study is that it does not have a control arm with manual compression.

Furthermore, no propensity matching was conducted as the results would not have been more meaningful, as the group size would have been small as a result.

## 7. Conclusions

Device failure is more common after using the extravascular MYNX Control system than after using the intravascular FemoSeal system. However, the need for surgical or interventional therapy due to device failure was low.

## Figures and Tables

**Table 1:** Baseline characteristics for the MYNX CONTROL and FemoSeal groups.

Baseline Characteristics	MYNX CONTROL *n* = 273	FemoSeal *n* = 275	*p*-Value
Age, yrs	72.7 ± 9.5	71.5 ± 11.1	0.089
Male sex	165 (60.4)	175 (63.6)	0.247
BMI	25.6 ± 4.5	25.8 ± 4.7	0.279
Hypertension	235 (86.1)	225 (81.8)	0.107
Diabetes mellitus	125 (45.8)	113 (41.1)	0.153
Hyperlipidemia	235 (86.1)	246 (89.5)	0.141
Smoker	87 (31.9)	100 (36.4)	0.154
Former Smoker	91 (33.3)	81 (29.5)	0.188
Coronary heart disease	129 (47.3)	109 (39.6)	0.043
PCI	94 (34.4)	85 (30.9)	0.215
CABG	27 (9.9)	21 (7.6)	0.217
CVD	57 (20.9)	63 (22.9)	0.319
Stroke	46 (16.8)	49 (17.8)	0.426
Renal insufficiency *	119 (43.6)	101 (36.7)	0.060
Dialysis	19 (7)	11 (4)	0.092

Values are *n* (%) or mean ± SD; BMI—body mass index; CABG—coronary artery bypass grafting; CVD—cerebral vascular disease; PCI—Percutaneous coronary intervention. * Defined as clearance < 60 mL/min.

**Table 2:** Pre-interventional laboratory and blood pressure.

	MYNX CONTROL *n* = 273	FemoSeal *n* = 275	*p*-Value
Creatinine (mg/dL)	1.44 ± 1.4	1.28 ± 1.2	0.073
Hemoglobin (g/dL)	13.1 ± 2.1	13.0 ± 2.2	0.277
INR	1.08 ± 0.22	1.07 ± 0.21	0.252
Thrombocytes (×10^3^/µL)	255 ± 82	272 ± 103	0.017
Systolic BP (mmHg)	144.8 ± 21.4	148.9 ± 24.8	0.020
Diastolic BP (mmHg)	76.9 ± 12.4	79.4 ± 14.2	0.014

Values are *n* (%) or mean ± SD; BP—blood pressure; INR—international normalized ratio.

**Table 3:** Pre-interventional medication for the MYNX Control and FemoSeal groups.

	MYNX CONTROL *n* = 273	FemoSeal *n* = 275	*p*-Value
Aspirin permanent medication	170 (62.3)	163 (59.7)	0.299
Aspirin loading dose	37 (13.6)	41 (15.1)	0.357
Clopidogrel permanent medication	86 (31.5)	77 (28.2)	0.227
Clopidogrel loading dose	181 (67.0)	176 (64.7)	0.315
Oral anticoagulation	92 (33.7)	89 (32.6)	0.428

Values are *n* (%).

**Table 4:** Puncture and intervention details.

	MYNX CONTROL *n* = 273	FemoSeal *n* = 275	*p*-Value
Puncture site (right)	158 (57.9)	143 (52)	0.097
Prior ipsilateral puncture	143 (55.2)	129 (50.6)	0.168
Prior use of a closure device	129 (49.8)	119 (46.7)	0.266
Prior use of a closure device in the last 3 months	25 (9.7)	23 (23)	0.462
Antegrade	157 (57.5)	101 (36.7)	<0.001
Introducer Sheath size 5	2 (0.7)	15 (5.5)	0.001
Introducer Sheath size 6	264 (96.7)	251 (91.2)	0.007
Introducer Sheath size 7	7 (2.6)	9 (3.3)	0.406
Lysis	5 (1.8)	14 (5.1)	0.031
Calcification CFA	170 (62.5)	179 (65.3)	0.275

Values are *n* (%); CFA—common femoral artery.

**Table 5:** Predictors of MYNX failure.

	OR	95%-CI	*p*-Value
Age (per year)	1.031	0.993–1.071	0.106
BMI < 25	1.639	0.822–3.266	0.160
BMI 25–30	0.504	0.239–1.064	0.072
BMI > 30	1.332	0.513–3.460	0.556
Female sex	1.281	0.642–2.557	0.483
Current smoker	0.733	0.339–1.585	0.430
Former smoker	1.197	0.587–2.442	0.621
Hypertension	1.078	0.393–2.960	0.884
Diabetes mellitus	0.841	0.420–1.681	0.624
Hyperlipidemia	0.454	0.195–1.054	0.066
Blood dialysis	0.712	0.158–3.215	0.659
Acetylsalicylic acid	1.369	0.658–2.849	0.401
Platelet aggregation inhibitors	0.869	0.409–1.845	0.715
(Non) vitamin K antagonist oral anticoagulants	0.398	0.168–0.944	0.036
Antegrade puncture	0.703	0.354–1.397	0.314
Retrograde puncture	1.423	0.716–2.828	0.314
Previous ipsilateral puncture	0.834	0.416–1.675	0.610
Previous closure device ipsilateral	0.947	0.472–1.901	0.879
Lysis	1.561	0.170–14.351	0.694
Calcification	1.644	0.869–3.111	0.126

BMI—body mass index; OR—odds ratio; 95%-CI—95%-confidence interval for the odds ratio.

**Table 6:** Predictors of FemoSeal failure.

	OR	95%-CI	*p*-Value
Age (per year)	0.981	0.938–1.026	0.397
BMI < 25	1.253	0.442–3.558	0.671
BMI 25–30	0.868	0.288–2.616	0.802
BMI > 30	0.799	0.174–3.669	0.772
Female sex	0.621	0.192–2.005	0.426
Current smoker	1.177	0.406–3.410	0.764
Former smoker	1.644	0.566–4.781	0.361
Hypertension	1.472	0.321–6.738	0.619
Diabetes mellitus	0.704	0.234–2.117	0.532
Hyperlipidemia	0.444	0.118–1.678	0.232
Blood dialysis	1.779	0.212–14.891	0.595
Acetylsalicylic acid	0.355	0.116–1.090	0.070
Platelet aggregation inhibitors	3.671	1.230–10.961	0.020
(Non) vitamin K antagonist oral anticoagulants	2.159	0.733–6.355	0.163
Antegrade puncture	1.158	0.400–3.353	0.787
Retrograde puncture	0.835	0.288–2.420	0.740
Previous ipsilateral puncture	2.034	0.675–6.127	0.207
Previous closure device ipsilateral	1.773	0.612–5.136	0.291
Lysis	3.179	0.644–15.708	0.156
Calcification	1.065	0.353–3.211	0.911

BMI—body mass index; OR—odds ratio; 95%-CI—95%-confidence interval for the odds ratio.

## Data Availability

No new data were created or analyzed in this study. Data sharing is not applicable to this article.

## Abbreviation

VCD—Vascular Closure Devices
